# Nonvolatile chirality switching in terahertz chalcogenide metasurfaces

**DOI:** 10.1038/s41378-022-00445-4

**Published:** 2022-09-30

**Authors:** Jiaxin Bao, Xieyu Chen, Kuan Liu, Yu Zhan, Haiyang Li, Shoujun Zhang, Yihan Xu, Zhen Tian, Tun Cao

**Affiliations:** 1 School of Optoelectronic Engineering and Instrumentation Science, Dalian University of Technology, Dalian, 116024 P. R. China; 2 Center for Terahertz waves and College of Precision Instrument and Optoelectronics Engineering, Tianjin University, and the Key Laboratory of Optoelectronics Information and Technology (Ministry of Education), Tianjin, 300072 P. R. China

**Keywords:** Micro-optics, Nanophotonics and plasmonics

## Abstract

Actively controlling the polarization states of terahertz (THz) waves is essential for polarization-sensitive spectroscopy, which has various applications in anisotropy imaging, noncontact Hall measurement, and vibrational circular dichroism. In the THz regime, the lack of a polarization modulator hinders the development of this spectroscopy. We theoretically and experimentally demonstrate that conjugated bilayer chiral metamaterials (CMMs) integrated with Ge_2_Sb_2_Te_5_ (GST225) active components can achieve nonvolatile and continuously tunable optical activity in the THz region. A THz time-domain spectroscopic system was used to characterize the device, showing a tunable ellipticity (from ‒36° to 0°) and rotation of the plane polarization (from 32° to 0°) at approximately 0.73 THz by varying the GST225 state from amorphous (AM) to crystalline (CR). Moreover, a continuously tunable chiroptical response was experimentally observed by partially crystallizing the GST225, which can create intermediate states, having regions of both AM and CR states. Note that the GST225 has an advantage of nonvolatility over the other active elements and does not require any energy to retain its structural state. Our work allows the development of THz metadevices capable of actively manipulating the polarization of THz waves and may find applications for dynamically tunable THz circular polarizers and polarization modulators for THz emissions.

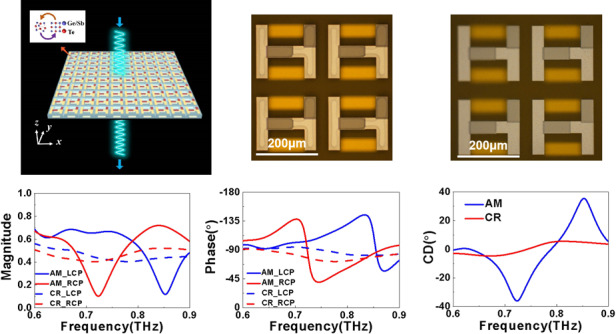

## Introduction

Chirality, referring to structures without any mirror symmetry planes, is ubiquitous in nature, ranging from polymers and molecules to crystals^1,2^. It plays a crucial role in medical and biological sciences since most biomolecules are chiral^3^. Nevertheless, naturally occurring chirality, such as circular dichroism (CD) and optical activity (OA), is very weak and requires bulk chiral materials to detect a chiroptical response^4,5^. Artificial chiral metamaterials composed of subwavelength components possessing electromagnetic (EM) resonances are the most appropriate platform for investigating chiral devices^6,7^. Their strong optical confinement, which produces intense light–matter interference, can overcome the weak chirality of naturally existing mediums. Chiral metamamaterials (CMMs) have been used for improving chiroptical responses, having various applications for wave plates^8^ and circular polarisers^9^. In the terahertz (THz) region, the modulation of CD and OA is extremely important since macromolecules with polar or ionic elements intensely absorb THz waves due to the existence of collective vibration modes^10,11^ and biopolymers, i.e., DNA, proteins, and RNA, consisting of chiral structures that selectively absorb circularly polarized light in the THz region^12^. Such features can be used to develop vibrational CD spectroscopy to verify molecular structures, such as chirality or tertiary structure^13,14^. In addition to this significance of chirality for the THz region, active polarization engineering of THz light may provide incredible potential for creating novel and transformative THz devices^15^. THz CMMs with large chirality values, particularly those endowed with tunability^16,17^, are highly efficient in manipulating the polarization of THz waves in a way that is far superior to other natural materials. This is of crucial importance for THz metadevices with sophisticated functions, such as external amplitude and polarization modulators in future THz wireless protocols^18^, exploration of the birefringence of a medium^19^ for pharmaceutical applications^20^ and stereochemistry^21^, and the rovibrational spectra excitation of inherently chiral complex chains^22^ and biological macromolecules^23^. Therefore, the integration of various active materials (e.g., silicon, superconductors, vanadium oxide, graphene, and liquid crystals) into conventional CMMs has been recently performed to enable active tunability via external optical or electrical stimuli^24,25^ capable of modulating the polarization state of THz light^17,26^. In particular, handedness-switchable THz CMMs using vertically deformable microelectromechanical systems (MEMS) have also shown promising applications for polarization modulation^27–29^. However, active modulation of the chiroptical response still faces formidable challenges, mostly owing to the limited multilevel response, volatility, and requirement for an extra pump laser and proper photonics kits^30,31^.

Phase change materials (PCMs) can offer a pronounced change in material properties over a wide spectral range during the structural state transition. As a representative of PCMs, germanium (Ge)-antimony (Sb)-telluride (Te) alloy^32^, which experiences an amorphous-to-crystalline state transition, has been intensively explored because of its potential applications in electronic and photonic devices. Different from the abovementioned active elements (e.g., vanadium oxide), the phase change of Ge-Sb-Te (GST) is steered by nucleation dynamics^33^. By continuously varying the proportion of its crystallization, one can attain an analog response rather than binary transition states. Crucially, these analog states are not volatile, which is based upon their ability to rapidly transition between amorphous and crystalline states through proper stimulus and keep the desired structural phase at room temperature^34^. Owing to the exceptional combination of rapidly reversible state transition and retention time^35^, GST has fueled both the nonvolatile data storage era^36^ and tunable photonics metadevices^37–41^. However, tunable metamaterials based on GST in the THz regime remain primarily unexplored, probably because of the lack of accessible optical characteristics of GST alloys in the THz region. Recently, it has been shown that GeTe can control THz waves^42^. Very recently, GST-hybridized THz metamaterials have been further studied for resonance transitions^43^. However, to date, the tuning of chiroptical responses (i.e., CD and OA) in GST-based metamaterials has not been investigated for the THz region.

In this work, we propose tunable THz CMMs by integrating Ge_2_Sb_2_Te_5_ (GST225) segments into a conjugated bilayer resonator. We experimentally demonstrate that through terahertz time-domain spectroscopy (THz-TDS) measurements, the chiroptical response can be switched on/off by transiting the structural state of GST225 between amorphous and crystalline. In particular, such an active CMM polarizer can obtain a switchable CD with an ellipticity modulation of ~36° and exhibit a polarization plane rotation of ~32° at 0.73 THz. Furthermore, the nonvolatile multilevel chiroptical response switching of states is experimentally obtained by modulating the crystallization ratios of the GST225 segments. The experimental measurement agrees with the numerical simulation, which attributes this efficient tunability to a variation in the conductivity of the GST225 components. GST225 has the advantage of a nonvolatile storage mechanism over the other active mediums; this means that GST225 only requires energy for the phase transition process and not for keeping a particular phase. Hence, once the CMMs are transited, they can maintain their chiroptical responses until they are transited again. This enables the chalcogenide CMMs to show promise from a green technology perspective. Given the strong switching response compared to natural materials, CMMs could find critical applications in THz technology, such as vibrational CD for identifying the chirality of organic molecule structures and providing accurate noncontact Hall measurements.

## Results and discussion

**Fig. 1:**
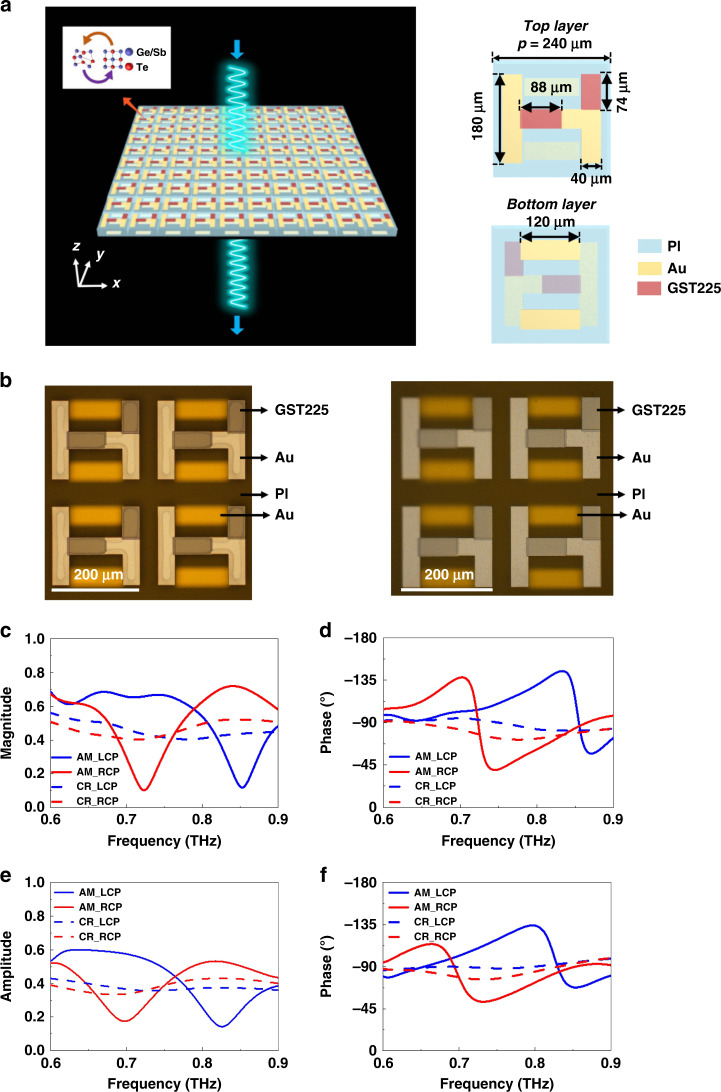
The demonstration of the tunable chiral metamaterials based on GST225. **a** Scheme of the chirality switching bilayer metamaterials based on GST225 strips. Inset: the meta-atom consisted of two different shaped resonators patterned on each side of the PI board. **b** Photos of the 2×2 resonator section of the fabricated metamaterials before crystallization (left column) and after crystallization (right column). Scale bar: 200 μm. The measured transmission **c** magnitude and **d** phase spectra of LCP (blue lines) and RCP (red lines) incident waves, with amorphous (solid lines) and crystalline (dashed lines) states. The simulated transmission **e** amplitude and **f** phase spectra of LCP (blue lines) and RCP (red lines) incident waves, with amorphous (solid lines) and crystalline (dashed lines) states

In Fig. 1a, we schematically show the layout of the conjugated bilayer metamaterials. Paired resonators with two different shapes are patterned on each side of the polyimide (PI) board. The H-shaped resonator on the front side of the PI interlayer was composed of both gold (Au) and GST225 strips, and the resonator on the backside was made of two parallel Au strips. The thicknesses of the Au and GST225 strips were *h*_*Au*_ = 0.1 μm and *h*_*GST*_ = 0.3 μm, respectively. The dimensions of the resonator are shown in the scheme of the meta-atom (right column of Fig. 1a). The pitch of the resonator array was *p* = 240 μm. The thickness of the PI interlayer was *h*_*i*_ = 40 μm. The geometrical parameters were optimized to enable the CMMs to switch on/off the CD response in the spectrum from 0.6 to 0.9 THz through the phase transition of GST225 between amorphous and crystalline. The complex permittivity of the PI dielectric interlayer was ε_r_ = 3.25 + 0.05i. We explain the working mechanism of the proposed chalcogenide metamaterials below. At room temperature of T_r_ = 25 °C, GST225 was in the amorphous state (left column of Fig. 1b); therefore, the GST225 strips did not affect the interference between the THz waves and Au strips. As a consequence, the conjugated bilayer resonator acts as a spiral G-shaped metallic structure, and intrinsic chirality can be expected. When increasing the temperature above T_c_ = 260 °C, the GST225 strips were fully crystallized, and the bilayer resonator worked as a Chinese character “sun”-shaped metallic meta-atom (right column of Fig. 1b). Thus, intrinsic chirality does not occur, owing to the axial symmetry of the Chinese character “sun”-shaped resonator. Hence, by switching the structural state of GST225 between amorphous and crystalline, the chiroptical response can be switched on/off. We optimized the structural geometries to excite the strong chiroptical effect in the THz region when the GST225 strips were in the AM state. The conjugated bilayer metamaterials were fabricated on a Si wafer. The proposed structure can be fabricated using a state-of-the-art microfabrication procedure (see Methods). The resonator array has an area of 1 × 1 cm, which is sufficiently large to satisfy the measurement requirement. In Supplementary Figure S1, we schematically demonstrate the fabrication process of the designed conjugated bilayer metamaterials. In the left column of Fig. 1b, we show a photo image of the conjugated bilayer metamaterials incorporated with the as-deposited amorphous GST255 strips. We then crystallized the amorphous GST225 strips by annealing the metamaterials on a hot plate. The photo images of the metamaterials with the crystalline states are shown in the right column of Fig. 1b. The control of optical activity was explored using a THz-TDS system (see Methods)^44^. The THz-TDS system offers 50-fs laser pulses with a repetition rate of 100 MHz, a spot diameter of 10 mm, and a central wavelength of λ = 1560 nm. The complex Jones matrices of the bilayer metamaterials were derived from experimentally measured data. The spectra of the transmission magnitude and phase were normalized by a bare Si wafer, the same as the metamaterial substrate. In Fig. 1c, we show the transmission magnitude spectra of the amorphous (solid lines) and crystalline (dashed lines) metamaterials under both left-handed circularly polarized (LCP) and right-handed circularly polarized (RCP) THz waves, respectively. The amorphous metamaterial intensely interferes with both LCP and RCP waves, but at two different frequencies. The transmission dips occur at 0.73 and 0.85 THz in the transmission magnitude spectra for RCP (solid red line) and LCP (solid blue line) incidences. When crystallizing amorphous GST225 via hot-plate annealing, the resonances for both LCP and RCP waves were wholly switched off and produced featureless transmission magnitude spectra. This modification in the transmission resonances was caused by increasing the conductivity of the GST225 strips, providing a considerable modulation depth and contrast ratio at 0.73 and 0.85 THz, respectively. The ultrasensitive characteristic of transmission resonance offers a vast modulation even for the slight increase in the conductivity of GST225. The phase modulators in the THz region were essential components for THz beam steering, beam shaping, holographic imaging, and controlling the polarization^45–48^. Nevertheless, the current phase handling techniques used in optical and microwave regions^49,50^ do not work effectively when employed at THz frequencies. Very recently, a GeTe-based coding metamaterial was demonstrated, showing its promising potential for controlling the phase of THz waves^51^. Herein, the alternation in the conductivity of GST225 under the different structural states was also employed for engineering the transmission phase response of metamaterials integrated with the GST225 strips. In Fig. 1d, we experimentally present the transmission phase spectra of the metamaterials with amorphous and crystalline states under both LCP and RCP incident THz waves. The state transition of GST225 between amorphous and crystalline can pronouncedly modulate the transmission phase for both LCP and RCP incidences. The metamaterials have a significant phase modulation of approximately 0.73 and 0.85 THz, respectively, when changing the structural state of GST225 from amorphous to crystalline. To further validate the effect of the state transition of GST225 on the spectra of the transmission magnitude and phase under the different circularly polarized waves, we have performed full-wave simulations to solve Maxwell’s equations for the bilayer metamaterials. In the simulation, the frequency-dependent conductivities of the GST225 film in the amorphous and crystalline states were determined by the THz-TDS measured data. Au was modeled as a lossy metal with a conductivity of σ_Au_ = 4.561×10^7 ^S/m. As seen in Figs. 1e and 1f, the simulated spectra of the magnitude and phase of chalcogenide metamaterials under the LCP and RCP incidences agreed well with the measured spectra (Figs. 1c and 1d). The geometry of the metamaterials is the same as that captured by the optical images shown in Fig. 1b. A commercial 3D full-wave solver (CST MICROWAVE STUDIO®) based on the finite integration technique was employed. We express the model in detail in the Methods. As presented in Fig. 1e, for the amorphous state, the transmission magnitude spectra of the RCP and LCP incidences showed intense resonances, with transmission dips at 0.7 and 0.83 THz, respectively. These two resonance frequencies were consistent with the measured frequencies, as illustrated in Fig. 1c. For the crystalline state, the resonances for both the LCP and RCP incidences were radically weakened. In Fig. 1f, the simulated spectra of the transmission phase under LCP and RCP incidences were also in excellent agreement with the experimental observations.

**Fig. 2:**
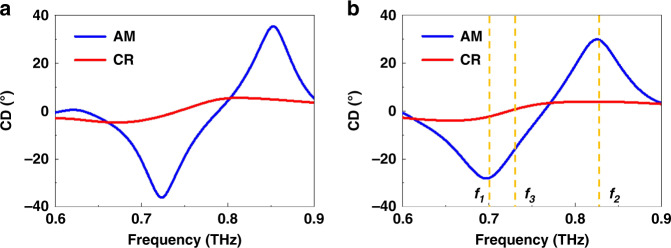
The switchable chiroptical response of the phase change metamaterials. The **a** measured and **b** simulated CD (ellipticity) spectra with the amorphous and crystalline states

CD is the most popular method for characterizing the optical activity of chiral materials. CD denotes the variation between the transmission spectra of two circular polarizations. Thus, it is associated with the ellipticity of the transmitted wave for a linearly polarized incidence, which is calculated by *θ* = arctan [(E_LCP_ – E_RCP_)/(E_LCP_ + E_RCP_)], where E_LCP_ and E_RCP_ represent the transmission magnitudes for LCP and RCP incidences, respectively^3^. As seen in Fig. 2a, the CD response can be switched on/off in the frequency range between 0.6 and 0.9 THz when transiting the GST225 state from amorphous to crystalline. The tunable CD response indicated that the metamaterials possessed chirality-switching behavior. The switchable chiroptical effect was robust and exhibited an angle variation of 30.5°. The numerically simulated CD spectra are shown in Fig. 2b. The CD simulations and the measurements were in good agreement, confirming the switchable chirality when varying the structural state between amorphous and crystalline. The slight difference between the measured and simulated data was probably caused by the existence of native oxides, rough surfaces, and defective fabrication.

In Supplementary Figure S2(a), the Raman spectroscopy setup is schematically illustrated. In Supplementary Figure S2(b), we present Raman spectra for 300 nm thick GST225 films with amorphous (blue line) and crystalline (red line) structural states. This analyses the structural phase of the GST225 film between amorphous and crystalline^52^.

For the amorphous state, three Raman peaks relating to the defective tetrahedral phonon modes were observed at 73, 125, and 150 cm^−1^ over a broad spectrum ranging from 60 to 200 cm^−1 ^^53^. Meanwhile, Raman spectroscopy illustrated that the crystalline GST225 layer had three bands resonating at 76, 105 and 172 cm^−1^ after 300 °C annealing. Compared to amorphous GST225, the Raman mode of 150 cm^−1^ disappeared, indicating that the GST225 film was fully crystallized by hot-plate annealing^54^. Supplementary Figure S2(c) experimentally shows the conductivity of the 300 nm thick GST225 film for the amorphous (blue line) and crystalline states (red line). The THz features of the GST225 film were characterized via a THz time-domain spectroscopy system (see Method). We extracted the conductivity from the THz time signal transmitted through the GST225 layer with the Si reference substrate. The measured conductivity of amorphous (blue line) and 300 °C hot-plate annealed (red line) GST225 layers shows vastly contrasting values. A large variation in the conductivity between the structural states can pronouncedly change the chiroptical resonances in the metamaterials. The change in conductivity was caused by bonding switching from predominantly covalent in the amorphous phase to resonant bonds in the crystalline phase^55^.

**Fig. 3:**
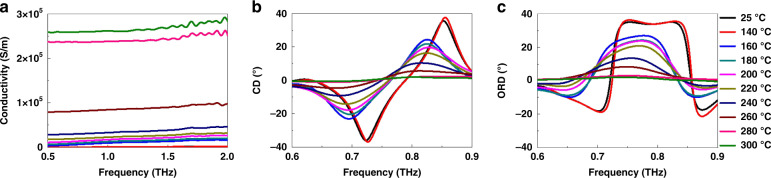
The conductivities of the GST225 layer and CD and c ORD at temperatures ranging from 25 to 300 °C. **a** The conductivities of the GST225 layer at temperatures ranging from 25 to 300 °C, where the THz-TDS system was used to measure the conductivity across a frequency range from 0.5 to 2 THz. The measured spectra of **b** CD and **c** ORD at temperatures ranging from 25 to 300 °C

It is noteworthy that our proposed structure provides on/off two-level switching by controlling the annealing temperature and has multilevel switching caused by the intermediate states (i.e., between purely amorphous and crystalline phases)^34^. Herein, the intermediate states were achieved via partial crystallization by modulating the annealing time at different hot-plate annealing temperatures. In particular, heat diffusion and the repeatable energy dose can control the partial crystallization process, enabling the cumulative transition of the GST225 layer^56,57^. As seen in Fig. 3a, a continuous change in the THz conductivity was obtained by engineering the crystallization ratio in the amorphous GST225 film by controlling the annealing temperature at a particular time. Furthermore, such intermediate states of GST225 were not volatile, thus not requiring power to maintain a certain state^33^. This characteristic is appealing for achieving continuously tunable THz devices. To show the function of the continuously tunable chiroptical response, a few more measurements were performed to engineer circular dichroism (CD) and optical rotatory dispersion (ORD) using partial crystallization in orthogonal GST225 strips integrated with bilayer metamaterials. The optical activity of chiral materials can be symbolized by CD and ORD, where CD refers to the variation in the transmission spectra between LCP and RCP incidences and ORD measures the polarization rotation angle stemming from the transmission-phase variation between the two circular polarizations. As seen in Figs. 3b, c, the strengths of both CD and ORD can be continuously decreased when gradually increasing the annealing temperature from *T*_c_ = 25 to 300 °C, showing a continuous enantiomeric switching function. It is evident that modulation of *T*_c_ can directly control the CD and ORD. In Supplementary Figure S3, we present the measured spectra of the transmission magnitude and phase for a linearly polarized THz wave at temperatures ranging from 25 to 300 °C. In Supplementary Figure S4, simulations of CD and ORD are carried out, and the characteristics of the experimental spectra are well replicated. The heating time was fixed at 2 min, while multilevel nonvolatile states could also be achieved by controlling the heating time^58^. Note that the state of GST225 can change from as-deposited amorphous to cubic crystalline at approximately 150 °C and then into hexagonal crystalline at approximately 300 °C^59,60^.

**Fig. 4:**
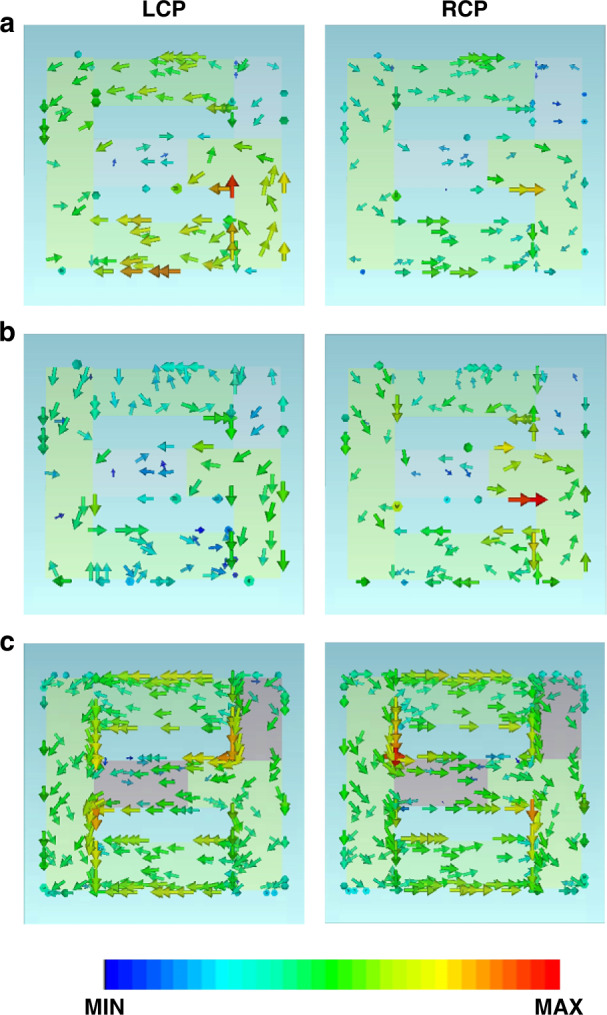
Simulated *J*_D_ distributions at the surface of the proposed bilayer metamaterials for LCP (left column) and RCP (right column) incidences. The *J*_D_ distributions at the frequencies of **a** *f*_1_ = 0.7 THz and **b** *f*_2_ = 0.83 THz, respectively, for the amorphous state. **c** The *J*_D_ distributions at the frequency of *f*_3_ = 0.73 THz for the crystalline state. These frequencies are marked in Fig. 1f. The arrows indicate the direction of *J*_D_

To explore the microscopic mechanism of the observed polarization response, we simulated the distributions of the surface current (J_D_) produced on the conjugated bilayer meta-atom with amorphous (Figs. 4a, b) and crystalline (Fig. 4c) states under illuminations of both the LCP (left column) and RCP (right column), respectively. For the amorphous state, at the first resonant frequency of *f*_1_ = 0.7 THz (as marked in Fig. 1f), it was shown that the surface currents were mainly distributed along the Au strips, whereas those along the orthogonal GST225 strips with the amorphous state were negligible (Fig. 4a). In this state, the proposed bilayer metamaterials made of an H-shaped resonator on the top layer and two parallel Au strips on the bottom layer can be treated as a spiral G-shaped metallic resonator, possessing significant intrinsic chirality^61^. The currents produced for the LCP wave appeared to exceed those for the RCP wave, leading to a considerable variation in the transmittance spectra of the metamaterials between the LCP and RCP waves. Such a variation in transmission magnitude produced a negative ellipticity; thus, the current distribution was consistent with the ellipticity spectra. For the second resonant frequency of *f*_2_ = 0.83 THz, similar relations were maintained for the J_D_ distributions, whereas the induced currents under LCP incidence were dominated by those for the RCP wave, producing positive ellipticity (Fig. 4b). For the crystalline state, at the frequency of *f*_3_ = 0.73 THz, it was found that the surface currents along the orthogonal GST225 strips were so intense that they were close to those along the Au strips (Fig. 4c). The structure can be treated as the Chinese character “sun”-shaped metallic metamaterials, which did not exhibit intrinsic chirality. Therefore, both the CD and ORD were negligible.

## Conclusion

In summary, we have experimentally illustrated a THz tunable chiral device based upon a bilayer metamaterial architecture integrated with GST225 active components. By switching the state of GST225 between amorphous and crystalline, an active switching on/off chiroptical response was achieved. In particular, continuous tunings of the ellipticity of 36° and ORD of 30° were recorded at approximately 0.73 THz, respectively. The experimental characterization was performed with a THz-TDS spectroscopy system and numerically validated with a 3D full-wave solver (CST MICROWAVE STUDIO®). Our work proposed a practical and compact THz polarization modulator, yielding the realization of fundamental optical components for THz imaging, polarization spectroscopy, and materials characterization.

## Methods

### Fabrication

The conjugated chalcogenide bilayer metamaterials were fabricated on a Si wafer. The Si wafer was first cleaned using acetone, isopropyl alcohol, and water in an ultrasonicator. A 5 μm thick PI layer was laminated to the Si wafer. The lamination procedure was performed through a standard office laminator with a temperature of 100 °C. The first layer of the paired Au strip array was fabricated on the surface of the 10 μm thick PI film. Photolithography was carried out to define the paired strip resonator array over an area of 1 × 1 cm. The 0.1 μm thick Au was thermally evaporated with a base pressure of 7 × 10^−6^ mTorr. Lift-off was used in acetone to obtain the Au paired strips resonators. A 40 μm thick PI layer was then spin-coated and thermally cured on the surface of the paired Au strip array. Subsequently, a 0.1 μm thick Au layer was deposited onto the 40 μm thick PI layer, followed by photoresist spin coating, photolithography, and lift-off, where the Au vertical cut-wire and L-shaped resonators were patterned. Finally, two orthogonal amorphous GST225 strips (0.3 μm thick) were deposited by radio frequency (RF) sputtering a GST225 alloy target onto the PI board, which created the profiles of an H-shape together with the Au vertical cut-wire and L-shaped resonators. We employed an RF power of 30 W and a chamber base pressure of 5 × 10^−6^ mTorr. A 20 nm thick SiO_2_ film was deposited onto the GST225 strips in the same chamber to prevent oxidation.

### Measurements

The switchable chiral metamaterial was experimentally characterized using THz-TDS spectroscopy. A low temperature-grown GaAs photoconductive antenna (iPCA, BATOP) was employed as the THz emitter, which can produce wideband THz waves illuminated by a femtosecond Ti: sapphire laser (Mai Tai, Spectra-Physics) with a repetition rate of *f* = 100 MHz and a central wavelength of λ = 1560 nm. The THz-TDS system has an employable bandwidth ranging from 0.1 to 2.0 THz. The transmissions of linearly polarized THz pulses through the bilayer metamaterial were measured in the time domain, with the amorphous and crystalline states. Herein, THz-TDS-based polarimetry measurements were performed to evaluate the complex Jones matrix of bilayer metamaterials. We input the linearly polarized THz wave pulses to the metamaterials and measured the polarization states of the THz wave pulses in the output. To obtain the transmission coefficients of CPL, which are *T*_+ +_, *T*_− +_, *T*_+ −_ and *T*_− −_, four linear copolarization and cross-polarization transmission coefficients were measured, *T*_*xx*_, *T*_*yx*_, *T*_*xy*,_ and *T*_*yy*_. The transmission coefficients of circularly polarized waves were then obtained from the linear measurements using the following equation:

1 $$\left( {\begin{array}{*{20}{c}} {T_{ + + }} & {T_{ + - }} \\ {T_{ - + }} & {T_{ - - }} \end{array}} \right) = \frac{1}{2}\left( {\begin{array}{*{20}{c}} {\left( {T_{xx} + T_{yy}} \right) + i\left( {T_{xy} - T_{yx}} \right)} & {\left( {T_{xx} - T_{yy}} \right) - i\left( {T_{xy} + T_{yx}} \right)} \\ {\left( {T_{xx} - T_{yy}} \right) + i\left( {T_{xy} + T_{yx}} \right)} & {\left( {T_{xx} + T_{yy}} \right) - i\left( {T_{xy} - T_{yx}} \right)} \end{array}} \right),$$

where the first and second subscripts denote the input and output THz waves, - and + denote the LCP and RCP THz waves, and *x* and *y* denote the two linearly polarized THz waves with the *E*-field polarized along two orthogonal axes. In Supplementary Fig. S5, we have schematically shown an experimental setup consisting of four polarizers, two lenses, a THz emitter and a detector. Polarizers 1 and 4 were placed horizontally. As seen in the inset, the polarization at +45° is defined as the X-polarization, and the polarization at −45° is defined as the Y-polarization. When polarizers 2 and 3 are both placed at 45°, the wave emitted through polarizers 1 and 2 becomes an X-polarized THz wave and is subsequently focused on the sample via lens 1. The X-polarized wave in the output of the sample can pass through polarizers 3 and 4, while the output Y-polarized wave cannot pass through polarizer 3. Therefore, the detector can only detect the transmitted X-polarized wave. (*T*_*xx*_) As polarizer 3 is changed to −45°, the output Y-polarized wave can pass through polarizers 3 and 4, while the X-polarized wave cannot pass through polarizer 3; therefore, the transmitted Y-polarized THz wave can be detected. (*T*_*xy*_) In contrast, by placing polarizer 2 at −45°, the THz wave radiating from the emitter can transmit through polarizers 1 and 2 and become the Y-polarized incidence. *T*_*yx*_ and *T*_*yy*_ can be experimentally obtained by setting polarizer 3 at +45° and −45°, respectively.

### Simulations

CST Microwave Studio was used to numerically calculate the transmission spectra for both LCP and RCP incidences. We employed unit cell boundary conditions along the *x-* and *y-*directions and adopted open and space conditions along the *z*-direction. We used a tetrahedral-type mesh in a frequency domain solver. The input THz emission was simulated with a top port radiating *E*_*y*_ polarized plane waves toward the metamaterials. Numerical simulations of the frequency-dependent complex permittivity of polyimide at THz frequencies were experimentally determined. Au was modeled as a lossy metal with a conductivity of σ_Au_ = 4.561×10^7 ^S/m. The THz-TDS system picked the frequency-dependent conductivities of the GST225 film in the amorphous and crystalline states measured data (see Fig. 2b).

## Supplementary information

Supplementary Information
