# Supplementary material for: Nonvolatile chirality switching in terahertz chalcogenide metasurfaces
Source: Microsyst Nanoeng. 2022 Sep 30;8:112. doi: 10.1038/s41378-022-00445-4 (PMC9525255; doi:10.1038/s41378-022-00445-4)
Supplement: Supplementary file 1 — Supplementary Information [file 41378_2022_445_MOESM1_ESM.docx]

Supporting Information

**Nonvolatile enantiomeric switching in terahertz chalcogenide metasurface**

Jiaxin Bao ^‡^, Xieyu Chen^‡^, Kuan Liu, Yu Zhan, Haiyang Li, Shoujun Zhang, Yihan Xu, Zhen Tian*, and Tun Cao*^,‡^

J.Bao, K. Liu, Y.Zhan, H.Li, Prof. T. Cao

School of Optoelectronic Engineering and Instrumentation Science

Dalian University of Technology

Dalian 116024, P. R. China

E-mail: [caotun1806@dlut.edu.cn](mailto:caotun1806@dlut.edu.cn)

X. Chen, S. Zhang, Y. Xu, Prof. Z. Tian

Center for Terahertz waves and College of Precision Instrument and Optoelectronics Engineering

Tianjin University, and the Key Laboratory of Optoelectronics Information and Technology (Ministry of Education)

Tianjin 300072, P. R. China

E-mail: [tianzhen@tju.edu.cn](mailto:tianzhen@tju.edu.cn)

Fig. S1 Scheme of fabrication processing of conjugated bilayer metamaterials integrated with the GST225 stripes.

Fig. S2 (a) Experimental configuration for Raman spectroscopy. We employ a spectrometer (Horiba iHR550) to collect and analyze the Raman scattering spectra under ~20 ^0^C. A 532 nm solid state laser was used to excite the structures. The power of the laser is 1.5 mW. The spectra were collected in the back-scattering geometry. A resolution of wavenumber is ~ 2.6 cm^-1^. The beam of the laser source was focused using a 100 × microscope with working distance of 0.2 mm. An air-cooled charge-coupled device (~70 °C) with a 1024 × 256 pixel front-illuminated chip was used to measure the scattering signal dispersed on a grating with 1800 grooves mm^-1^.

**(b)** Raman, and **(c)** THz conductivity spectra of the GST225 film—amorphous (blue line) and crystalline states (red line).

Fig. S3 The measured spectra of transmission magnitude (left column) and phase (right column) for linear polarized THz T*_xx_* (black lines), T*_xy_* (red lines), T*_yx_* (blue lines), and T*_yy_* (green lines) at the various temperatures of (a) 25 ℃, (b) 140 ℃, (c) 160 ℃, (d) 180 ℃, (e) 200 ℃, (f) 220 ℃, (g) 240 ℃, (h) 260 ℃, (i) 280 ℃, and (j) 300 ℃.

Fig. S4 The simulated spectra of (a) CD and (b) ORD at the temperature ranging from 25 to 300 ℃.

Fig. S5 The scheme of experimental setup consisting of four polarizers, two lenses, a THz emitter and a detector.
